# Social, Community, and Cultural Factors Associated with Parental Stress in Fathers and Mothers

**DOI:** 10.3390/ijerph20021128

**Published:** 2023-01-09

**Authors:** Camilla K. M. Lo, Mengtong Chen, Qiqi Chen, Ko Ling Chan, Patrick Ip

**Affiliations:** 1 Department of Applied Social Sciences, The Hong Kong Polytechnic University, Hung Hom, Hong Kong, China; 2 Department of Social Work, The Chinese University of Hong Kong, Shatin, Hong Kong, China; 3 Department of Social Work, School of Sociology and Anthropology, Xiamen University, Xiamen 361005, China; 4 Department of Paediatrics & Adolescent Medicine, The University of Hong Kong, Pokfulam, Hong Kong, China

**Keywords:** parenting, parenthood, gender differences, working-class families

## Abstract

Parenting stress is a key factor in predicting the quality of parent–child relationships and child development outcomes. Previous research tends to focus on examining individual factors contributing to parental stress, with minimal attention to other important contextual factors that may affect parenting. This study examines the issue from a broader ecological perspective by investigating social, cultural, and community factors associated with parental stress in a community sample of economically active fathers and mothers in Hong Kong. A secondary analysis was conducted using the data from the 2017 Family Survey, a territory-wide household survey conducted in Hong Kong. The data of the current study included a sub-sample of 736 working-class parents (48.4% males and 51.6% females). The mean age of fathers and mothers was 50.99 (*SD* = 11.2) and 48.68 (*SD* = 10.34) years, respectively. Mothers reported significantly higher levels of parental stress than fathers, *t* = −4.241, *p* < 0.001. Different social, cultural, and community factors were associated with parental stress for fathers and mothers. Strong endorsement of traditional family values (*B* = −0.23, *p* = 0.032) and frequent practice of filial piety (*B* = −0.005, *p* = 0.019) reduced parental stress in fathers. Additionally, fathers who perceived formal support as effective scored higher levels of parental stress, *B* = 0.20, *p* < 0.001. For mothers, informal social support from family members was the only social predictor for reduced parental stress (*B* = −0.14, *p* < 0.001) among all the other contextual variables. Community support to reduce parental stress in working parents should address the respective risk factors for fathers and mothers.

## 1. Introduction

Demands associated with the role of parenting and childrearing oftentimes result in the experience of psychological distress, which refers to parental stress [1,2]. Decades of research have confirmed the association between elevated parental stress and parental mental health conditions and quality of life [3,4]. There are also a wide range of adverse consequences of parental stress on children’s developmental and behavioral outcomes [5,6], such as internalizing and externalizing problems [7,8], and impaired social cognition and competence [9,10]. In parallel, there is a large body of literature substantiating the impacts of parental stress on the interaction and communication between parents and children [11]. It is found that parents with higher levels of parental stress are less affectionate and involved with their children [12], which in turn leads to deteriorated quality of the parent–child relationship [13,14]. More importantly, prior studies have indicated a positive link between parental stress and child abuse and neglect [15], which is a prevalent social and public health problem across the globe. These findings emphasize the importance of immediate efforts to alleviate parenting-related stress for parents.

Based on ecological models of parenting, parental stress is influenced by a number of interrelated variables at different levels, ranging from individual characteristics of parents and children, family, and community factors, to the broader socio-cultural context [16,17]. With respect to parent-related predictors, consistent evidence has shown that a lower family income and education level [18,19], impaired mental health, and childhood trauma [20,21,22] are positively associated with parental stress. In the same vein, ample studies have focused on the influence of child-related characteristics on parental stress. Several meta-analyses provide evidence that parents of children with developmental disorders [23,24] and chronic physical conditions [25] perceive significantly more parental stress than those of typically-developing children. Meanwhile, a large number of studies have explored the impacts of family characteristics, such as instability and complexity of family arrangements [26] and impaired family functioning [18] on parents’ levels of stress. In sum, exhaustive research has been done to identify a myriad of predictors of parental stress at the parent, child, and family levels.

In light of the heavy emphasis on individual and family factors in this line of research on parental stress, most of the existing interventions aim at modifying parent, child, and family characteristics to mitigate the stress associated with parenting, leaving aside community and socio-cultural variables [27]. Given that there are individuals and families who do not reach out for help due to having limited access to services and stigmatization [28], individual- and family-focused interventions may not be sufficient to address the condition. As such, it is crucial to pay more attention to investigating variables associated with the contexts in which parents are situated, including the social, community, workplace, and cultural environments to inform the development of community-based support and services for parents in a more holistic manner. Among the scant literature evaluating parental stress through this broader lens of social and community influences, it is found that social support, social connections, and governmental support are protective factors against high levels of parental stress [22,29,30]. Apart from social and community influences, parental stress is affected by the extent to which one’s cultural values are congruent with the environment [31]. For example, in the United States, Asian mothers reported higher levels of parental stress than Caucasian mothers, potentially due to differences in culturally related parenting values and structural disadvantages [32]. Additionally, the endorsement of traditional family values and beliefs on marriage and childrearing is correlated with one’s experience of parental stress. Specifically, a Chinese study revealed that intact family and dual parenting are highly valued while the social stigmatization of divorce as unacceptable and shameful still persists in contemporary societies [33], which potentially leads to a rise in parental stress among single parents. Taken together, results from these studies illuminate the importance of considering social, community, and cultural variables to inform the development of culturally responsive and appropriate services to reduce parental stress at the community level.

Another salient limitation emanating from the previous endeavor concerns the study samples. Although research has consistently found elevated levels of parental stress among parents in different clinical groups, such as parents of children with developmental disorders and chronic illnesses [23,24,25], the predictors of parental stress for the general parent population, especially working parents, are understudied [34,35]. A recently published systematic review has explored 29 studies examining factors associated with parenting stress in general parents [36]. Surprisingly, none of the studies reviewed looked into parenting stress among employed parents in the general population, further indicating that there is little research in this area. Working hours in Hong Kong are among the highest in the world, with an average of 43 working hours per week, and 30% of the working population reported excessive working hours of 49 or more per week [37]. Considering the extended working hours and demand from the workplace, dual-career parents are found to experience high levels of role strain and distress in Hong Kong [38,39]. Interestingly, a recently published article using data from a territory-wide household survey in Hong Kong showed that this kind of distress equally affected fathers and mothers [40]. As such, additional work-related factors of parental stress, such as work satisfaction and the demand of maintaining a balance between work and family life, would add another layer to one’s perceived level of parental stress and are worthy of investigation within the ecological model. In addition, findings regarding the gender differences in parental stress are somewhat inconclusive about whether fathers and mothers experience stress differently [41,42], thus warrant additional investigation.

Building on the past literature that parental stress reflects a multifaceted and multileveled influence rather than an individual factor, the present study adopted an ecological approach by examining social, community, and cultural factors associated with parental stress among working fathers and mothers in Hong Kong, using a non-clinical, heterogeneous community-based sample.

## 2. Materials and Methods

### 2.1. Study Design and Sample

Using data from the 2017 Family Survey, a territory-wide household questionnaire, a secondary analysis was conducted to investigate various predictors of parental stress at the social, community, and cultural levels among economically active parents in Hong Kong. The Family Survey was carried out by the Family Council, an advisory body to the Hong Kong government, on a biannual basis beginning in 2011 [43]. These surveys provided updated and empirical information regarding the changes and development among Hong Kong families in the following domains: the importance of family, parenthood, family functioning, satisfaction with family life, work–family balance, the availability of social support networks, and the awareness of and participation in family-related programs. The 2017 Family Survey [44] covered all persons aged 15 or above residing in Hong Kong as the target population. A two-stage stratified random sampling method was adopted to recruit participants. A total of 6500 living quarters were randomly sampled from the Frame of Quarters maintained by the Census & Statistics Department, as stratified by geographical area and type of quarter. In the second stage, a household member aged 15 years or above in each household was randomly selected for completing the survey using the last birthday method. The 2017 Family Survey recruited an effective sample of 3000 persons, in which 48% of the respondents were employed. The data of the current study included a sub-sample of the 2017 Family Survey. Among the 48% of participants who were employed or economically active, those who ever had a child were included in the present analysis. The selected sub-sample consisted of 736 working parents (356 fathers and 380 mothers) from different families. 

### 2.2. Measures

#### 2.2.1. Demographics

Several demographic variables, including age, education level, marital status, average monthly personal income, as well as the average number of working days and hours per week, were collected. Education attainment was coded as primary education or lower, secondary education, and post-secondary education or above. Marital status was coded as married or cohabiting with a partner, divorced or separated, never married, and widowed.

#### 2.2.2. Outcome Measures

Parental stress was assessed using 10 items. The 10 statements describe different aspects of stress that may arise since the child was born and respondents were asked to indicate their level of agreement with each item on a 5-point Likert scale, ranging from 1 (strongly disagree) to 5 (strongly agree). The statements included “more tired than before”, “having to spend most of my time fulfilling my child(ren)’s needs”, “having no private time”, “feeling overwhelmed when dealing with the issues related to my child(ren)”, “having more conflicts with my partner”, “no one provides help when I am in need”, “my family encounters financial difficulties”, “having a better relationship with my partner”, “talking more with other parents regarding the parenting skills”, and “happier than before”. The mean of the items was computed, with a higher score representing higher levels of stress. Cronbach’s alpha of the measure was 0.74.

#### 2.2.3. Predictors

Social and work factors were assessed by examining the availability of assistance from family members, the level of stress resulting from efforts to meet the competing demands of work and family life, and satisfaction with work life. Six items were used to assess parents’ perceived social support networks, specifically the availability of assistance from family members under six circumstances, including “when you are sick”, “when you need to make an important decision”, “when you are depressed and upset”, “when you are unemployed and cannot get a job”, “when you have financial problems”, and “when you want to share your happiness with your family members”. Respondents rated the global level of helpfulness and support they received in these situations on a 6-point Likert scale, scoring from 1 (not helpful or supportive) to 6 (helpful or supportive). A higher mean score indicated more helpful and supportive assistance from family members. The measure had a Cronbach’s alpha of 0.92. The level of stress resulting from efforts to meet the competing demands of work and family life was constructed as a one-item variable, in which respondents were asked to rate their level of stress in regard to balancing work and family life in general on a 4-point Likert scale, with 1 indicating no stress at all to 4 indicating a great deal of stress. A higher score suggested higher levels of stress experienced due to the demands of maintaining work and family life balance. In addition, respondents were asked to rate their overall satisfaction with work life on a single item, from 1 (very dissatisfactory) to 5 (very satisfactory).

Endorsement of cultural values was assessed by examining traditional attitudes toward family roles, endorsement of traditional family values, and the practice of filial piety. The index regarding traditional attitudes toward family roles consisted of three items, namely “men and women are expected to contribute to family income”, “fathers are financial providers while mothers are family caregivers”, and “men and women are both responsible for housework and daily chores”. Respondents were asked for their level of agreement with each statement on a 5-point Likert scale, from 1 indicating strongly disagree to 5 indicating strongly agree. Respondents with a higher mean score held more traditional attitudes toward family roles. The Cronbach’s alpha of the measure was 0.22. Seven items were used to probe views with respect to traditional family values: “having a son to continue the family name”, “having a son is better than having a daughter”, “family disgrace should be kept within the family”, “work hard to bring honor to the family”, “seek elder’s help to resolve family conflict”, “it is easy for daughter-in-law and mother-in-law to meet each other, but difficult for them to live well together”, and “consult parents for major decisions”. Each item was rated from 1 (strongly disagree) to 5 (strongly agree) on a 5-point Likert scale. Respondents with a higher mean score had a stronger endorsement of Chinese family values. The Cronbach’s alpha was 0.64. Six practices of filial piety, namely caring, respecting, greeting, pleasing, obeying, and providing financial support, were measured. Respondents were asked to report the frequency of engaging in these six practices towards their parents in the past three months. Items were rated on a 5-point Likert scale, where 1 = very little to 5 = very much. The filial piety scores were then calculated as a composite of these practices using a scale of 0 (very little); 25 (rather little); 50 (average); 75 (rather a lot); and 100 (very much). A higher mean score indicated more frequent filial piety. The measure had a Cronbach’s alpha of 0.87.

Community factors were assessed by examining family-friendly policy, whether or not the participant was aware of family-related activities organized by the Government or other organizations, and perceived effectiveness of family counselling and family education services. The availability of family-friendly policies was evaluated dichotomously. Respondents determined whether 10 different policies were implemented (1 = present and 0 = absent) at their workplace. Examples included five-day workweeks, compressed workweeks, flexible work schedules and arrangements, and paternity leave. A higher mean score indicated the presence of more family-friendly policies. One question item asked about whether or not respondents were aware of the family-related promotional activities or programmes organized by the Government and/or non-governmental organisations. Formal support was assessed by four items measuring parents’ perceived effectiveness of family counseling and family education services, in terms of relieving emotional distress related to family members, handling family problems, enriching knowledge of societal/community resources, and enhancing understanding among family members. Respondents were asked to rate each of the four items on a 5-point Likert scale, ranging from 1 (ineffective) to 5 (effective). A higher score meant respondents perceived family counseling and education services as being more effective. Cronbach’s alpha was 0.95.

### 2.3. Ethics Approval

The study involved secondary data analysis, and ethics approval was approved by the Home Affairs Bureau of the Government of the Hong Kong Special Administrative Region.

### 2.4. Data Analysis

Descriptive statistics were used to summarize participants’ demographic characteristics, including age, education attainment level, marital status, average monthly household income, and weekly average number of working days and hours. Descriptive data were also calculated to analyze the level of parental stress and the predictor variables, namely the availability of assistance, the level of stress resulting from meeting the demands of work and family life, work satisfaction, attitudes toward family roles and traditional values, the practice of filial piety, family-friendly policy, and the effectiveness of formal support. Further, parental stress level and all the predictor variables were compared between fathers and mothers using chi-square tests and t-tests to examine any potential gender differences. Next, a series of hierarchical regression analyses were performed to estimate the effects of the social, cultural, and community factors on parental stress outcomes while controlling for demographic characteristics. This approach enabled the consideration of the relative contributions of each domain of predictors. In the first set of regression analyses, demographic characteristics were entered as independent variables to control for these previously established parent-level predictors of parenting stress. Social and work, cultural, and policy/community factors were added as higher-level predictors, respectively, in the following three regression analyses in an additive and gradual manner. The final multivariable regression model included variables in all three domains. Parental stress remained as the dependent variable in all models. The same set of analyses was conducted separately on fathers and mothers to evaluate gender differences in the associations of the contextual factors with parental stress. Listwise deletion was used to handle the missing data in the regression analyses. Statistical analyses were completed using the Statistical Package for Social Science (SPSS, version 25.0) and the significance level was determined by two-tailed tests with a *p*-value less than 0.05 deemed significant.

## 3. Results

### 3.1. Sample Characteristics

Table 1 summarizes the demographic characteristics of the respondents. Among the 736 eligible parents who completed the survey, 48.4% (*n* = 356) were males and 51.6% (*n* = 380) were females. The mean age of the fathers and mothers was 50.99 (*SD* = 11.2) and 48.69 (*SD* = 10.34) years, respectively. A total of 66.8% of the overall sample completed secondary education while 19.2% received primary education and 14% attained tertiary education. Regarding marital status, the majority of parents (81%) were married or cohabited with a partner. A monthly personal income of less than HKD 29,999 (approximately USD 3863) was reported by 82.3% of the sample. Respondents worked 5.43 days (*SD* = 2.22) and 44.57 h (*SD* = 14.74) per week on average.

### 3.2. Comparison between Fathers and Mothers

Descriptive statistics and results of the chi-square and t-tests comparing parental stress and all predictor variables between fathers and mothers are presented in Table 2. The *t*-test indicated significant gender differences in perceived parental stress, *t* = −4.241, *p* < 0.001, with mothers reporting higher levels of parental stress (*M* = 3.03, *SD* = 0.46) than fathers (*M* = 2.88, *SD* = 0.46). With respect to the contextual predictors, significant differences were only found in traditional attitudes toward family roles, *t* = −3.995, *p* < 0.001, and the practice of filial piety, *t* = −3.150, *p* = 0.002 between fathers and mothers. Mothers tended to have a stronger endorsement of egalitarian attitudes toward family roles (*M* = 3.53, *SD* = 0.41) and engaged in more frequent practice of filial piety (*M* = 66.66, *SD* = 15.4).

### 3.3. Contextual Predictors of Parental Stress for Fathers and Mothers

To assess whether the social and work, cultural, and community factors would explain significant variance in parental stress and identify gender-specific predictors of parental stress, a series of multiple regression analyses were completed separately for fathers and mothers (see Table 3). For fathers, several demographic characteristics, including young age (*B* = −0.012, *p* < 0.001), low education level (*B* = −0.033, *p* = 0.044), and divorce/separation/widower (*B* = 0.311, *p* = 0.001) were significantly associated with higher parental stress in model 1. In model 2, none of the social and work-related factors were significantly associated with parental stress. When the cultural factors were included in model 3, fathers who had a stronger endorsement of traditional family values (*B* = −0.230, *p* = 0.032) experienced lower levels of parental stress. The association between filial piety practice and parental stress was significant but weak in strength (*B* = −0.005, *p* = 0.019). Results of the final model revealed that the contextual factors together accounted for a significant proportion of variance in parental stress, *R*^2^ = 0.224, *F*(14, 181) = 3.432, *p* < 0.001. Perceived effectiveness of family support services was the only significant policy/community determinant of parental stress, in which fathers who perceived family supportive services as effective reported higher levels of parental stress, *B* = 0.202, *p* < 0.001.

For mothers, marital status was a significant predictor of parental stress in model 1. Mothers who were divorced/separated/windowed perceived more parental stress, *B* = 0.131, *p* = 0.031. Social and work factors were added to model 2 and informal social support was found to be significantly associated with parental stress (*B* = −0.139, *p* < 0.001), in that mothers who received more support from family members scored lower levels of parental stress. Analysis in model 3 revealed insignificant associations between endorsement of cultural values and parental stress. The overall model was significant and the included predictors explained a significant amount of variance in parental stress, *R*^2^ = 0.162, *F*(14, 219) = 2.817, *p* < 0.001. None of the policy/community factors were significantly associated with parental stress for mothers.

## 4. Discussion

### 4.1. Study Findings

The current study expands the literature base on parental stress by identifying social, cultural, community, and gender-specific correlates from an ecological perspective, using a community-based sample of working-class parents in Hong Kong. Significant gender difference was found in the level of parental stress, which is consistent with the results of previous studies that mothers generally report higher levels of parental stress in comparison with fathers [18,35,42,45]. This finding corroborates the notion that contemporary families perpetuate the deep-seated gender role beliefs that mothers are the primary caregivers who undertake parenting and childrearing responsibilities [46,47], thereby resulting in high parental stress levels. Despite the increasing involvement of fathers in childcare [48,49], it is suggested that men tend to view family labor as optional owing to their major role as breadwinners, which protects them from experiencing high levels of parental stress [50]. Another explanation for the finding is that parental experience is shaped by the types and contexts of childcare activities in which parents engage [51]. Mothers usually feel burdened by task-focused care that encompasses instrumental duties, while fathers are more likely to engage in recreational activities with children [50,51]. This highly gendered parental involvement may contribute to the disparities in stress between fathers and mothers in the study sample.

With regard to the gender-specific factors, there was evidence confirming that different factors predicted parental stress for fathers and mothers. In line with the existing studies, fathers with certain demographic characteristics, including young age [52], lower education level [18,29], and divorce/separation [23], experience more parental stress. Additionally, strong endorsement of traditional family values and practice of filial piety were identified as significant cultural predictors of reduced parental stress for fathers, implying that cultural characteristics serve a protective function against psychological stress. The result of the protective function of strong endorsement of traditional family values partially contradicts the finding of past research that egalitarian attitudes toward gender roles reduce parenting stress in fathers [42]. Taking into consideration the Hong Kong context, there has been public education and policy promoting gender equality, but this ideology is not necessarily carried out in daily practice [53]. Hence, it is likely that fathers who strongly endorse traditional family values tend to have a higher sense of superiority over other family members, viewing caregiving in the family as women’s responsibility [53,54]. This perception may result in a lower caregiving burden, thus an experience of lower parental stress. Regarding filial piety as another protective factor, it may be the case that filial piety alters one’s appraisals of the caregiver role, which in turn indirectly influenced the caregiving burden [55]. The core concept of filial piety highlights interdependence and reciprocity within a family. In Hong Kong, a recent study revealed that males generally held a more positive attitude toward grandparental involvement in childcare [56]. Therefore, fathers who practice filial piety may be more open to receiving support from grandparents and experience less caregiving stress. However, it should also be noted that the magnitude of the association between filial piety and parental stress is small, thus additional research to verify this finding is needed.

One community factor uniquely associated with higher levels of parental stress for fathers was the perceived effectiveness of counseling and family education services. It is possible that fathers who experienced higher levels of parental stress tended to seek help from social services, hence viewing these services effective. In contrast, fathers who did not experience high levels of stress may not seek help from services, therefore seeing the services as less effective. However, as the study did not ask fathers to report their actual utilization of the family support services, such speculation cannot be ascertained based on the data of the current study. Given that several barriers hindering fathers’ help-seeking behaviors and participation in support programs have been documented, such as a mismatch between formal support programs offerings and fathers’ needs and desires, and the lack of father-friendliness in the service settings [57], future research work looking closely into how actual service utilization, help-seeking barriers, and perceived usefulness of services are associated with fathers’ parenting stress will provide useful information for services design.

Regarding the parental stress predictors for mothers, marital status is consistently found to be a significant predictor of parental stress in the published literature [20,24]. In light of the dominant cultural discourse on marriage, social stigmatization of divorce [32], coupled with other stressors associated with divorce, such as financial strain [58], emotional distress due to separation [59,60], and time responsibilities [61] potentially explain the increase in parental stress for single mothers. Among all the contextual domains, assistance from family members stood out as the only social factor significantly related to lower levels of parental stress. This coincides with prior studies that social support is negatively associated with parental stress [24,28,29]. Taking the Chinese context into consideration, extended family members, in particular grandparents, play an important role as complementary childcare providers in Hong Kong’s dual-earner families [48,62]. This fits with previous findings that emotional and instrumental support for childrearing provided by grandparents buffers parents’ risk of psychological distress [63,64]. In contrast, the perceived effectiveness of formal services did not predict maternal parenting stress. Past literature suggests the opposite, that formal parenting support, such as advice on child problems from professionals and childcare assistance provided by family centers, mitigates parental stress of mothers of children with developmental disabilities, who usually have higher caregiving demands and encounter more child-related problems [61]. Nevertheless, our non-clinical, heterogeneous, working-class mothers might not find formal support helpful in reducing parenting stress, while general and informal support better matched their childrearing needs.

Surprisingly, our study yielded different results from existing studies regarding how family-friendly policies in the workplace are related to parental stress. While there is evidence of the negative effects of the lack of family-friendly arrangements at work and workplace inflexibility on parental stress [35,65], we found no relationship between the availability of family-friendly policies in the workplace and parental stress for either fathers or mothers. A possible explanation for this finding is that the adoption of family-friendly employment practices in Hong Kong is very limited, as illustrated by the descriptive statistics showing that only an average of two types of family-friendly policy were reported by the participants. Additional research to clarify the association between family-friendly policy and parental stress is needed.

### 4.2. Limitations

Findings of this study should be interpreted within the context of its limitations. First, the measurements used were either single-item or items constructed by the Family Survey, although there are standardized and validated scales for measuring the predictors such as the Parenting Stress Index [66]. This limitation is a result of the use of secondary data from a large-scale survey. The use of a single and global item to capture participants’ perceptions and health status has been common in large-scale surveys which consider respondent burden and the costs of data collection [67]. Relatedly, the Cronbach’s alphas of some self-constructed measures such as traditional attitudes towards family roles and values were low, which may be due to the low number of items and heterogeneity nature of the constructs. Second, the reliance on self-report was subject to social desirability bias or inaccurate reporting. Specifically, social desirability bias may be more likely to occur when parents respond to questions related to parenting stress [68], resulting in a tendency to under-report high levels of stress. Third, the study utilized data from a cross-sectional survey, meaning no conclusion could be drawn about the causal directions of the relationships identified. Future research with a longitudinal design is needed to delineate the temporal relationships of the contextual factors for parental stress. Fourth, another limitation of the study is the relatively high mean age of the parents, which may be in part due to the increasing trends in marriage postponement and fertility rates in older age groups in Hong Kong [69,70]. The findings of the study may not be generalizable to parents of younger age. Moreover, as the selection of participants was based on whether they ever had a child, not based on the child’s age, the sample might include parents of children of very different ages, including adult children. Hence, the findings reflect the overall parental stress condition among parents in general, rather than a specific group of parents. Last but not least, the study was unable to factor in other potential confounding factors, such as the number of children per family, their gender and age, employment status of another parent (if present), parental health and mental health conditions, which might lead to inaccurate estimations of the effects of the predictors on parental stress in the analyses. Furthermore, the study relied on the participants’ self-report on the characteristics of different contextual factors, which is subject to biases. In future, researchers might consider collecting multiple sources of data and adding the aforementioned variables to the regression models in order to yield more rigorous results.

### 4.3. Implications

The results of our study offer several practical implications and open lines of inquiry for future research. In light of the gender difference in predictors associated with parental stress, researchers and practitioners are encouraged to tailor supportive family services and interventions for fathers and mothers, respectively. Supportive services that address the factors associated with parental stress would be desirable. Specifically, mothers may benefit from programs that emphasize and facilitate informal social support from family members, such as grandparents. Although the results showed that fathers’ perceived effectiveness of family counseling and family education services was related to higher levels of parental stress, it should not be interpreted as a casual effect and assumed that formal support is unhelpful for fathers. Based on the study’s findings, it is suggested that community services such as public education addressing cultural values influencing parental stress may be beneficial for fathers.

In terms of future research directions, clarification on the underlying mechanisms regarding the impact of cultural values on parental stress for fathers and mothers will deepen our understanding of the manifestation of the broader socio-cultural influences within families. In particular, it remains inconclusive whether traditional versus egalitarian views on family beliefs and practices contribute to better psychological well-being and adjustment for fathers. Parental involvement likely plays a role in explicating the discrepancies, and further work is warranted to decipher these convoluted relationships. Moreover, given that the regression models of the present study only account for 16% and 22% of the variance in parental stress in mothers and fathers, respectively, we call for more research efforts to examine other social, cultural, and community factors underlying this stress. Such understanding will inform the development of prevention and intervention strategies to address parental stress at the community and cultural levels.

## 5. Conclusions

Using a community-based sample of economically active parents in Hong Kong, the present study demonstrates that different social, cultural, and community factors predict parental stress for fathers and mothers. Informal assistance from family members reduces mothers’ stress in childrearing. Fathers who display strong support for traditional family values and practice filial piety frequently find parenting less stressful. However, the perceived effectiveness of formal family services is associated with elevated parental stress for fathers. The findings highlight the importance of gender-specific family support programs and services to be effective in addressing parental stress. An intervention approach capitalizing on informal social support from family members may be suitable for mothers, while programs addressing cultural values will alleviate parenting stress in fathers. A consortium of community and social efforts in addressing parental stress will not only have positive impacts on parents, but also children’s development and health outcomes.

## Figures and Tables

**Table 1:** Demographic characteristics of the participants.

	Total (*n* = 736)	Fathers (*n* = 356)	Mothers (*n* = 380)
Age (*M, SD*)	49.80 (10.82)	50.99 (11.20)	48.69 (10.34)
Age range	18–84	18–84	18–75
Educational attainment			
Primary education or lower	19.2%	18.0%	20.3%
Secondary education	66.8%	67.4%	66.2%
Post-secondary education or above	14.0%	14.6%	13.5%
Marital status			
Married/cohabiting with a partner	81.0%	87.9%	74.4%
Divorced/separated	9.1%	5.3%	12.7%
Never married	4.4%	4.8%	4.0%
Widowed	5.6%	2.0%	9.0%
Monthly income (individual)			
$9999 or below	23.2%	9.3%	36.3%
$10,000–$19,999	42.7%	43.8%	41.6%
$20,000–$29,999	16.4%	24.4%	8.9%
$30,000–$39,999	1.0%	1.4%	0.5%
$40,000–$49,999	5.8%	8.1%	3.7%
$50,000 or above	1.1%	1.7%	0.5%
No information provided	9.8%	11.2%	8.4%
Average no. of work hours per week	44.57 (14.74)	48.42 (13.68)	40.95 (14.81)
Work hour range	15–154	8–154	5–91
Average no. of work days	5.43 (2.22)	5.64 (3.02)	5.24 (0.97)

**Table 2:** Descriptive statistics of the study variables.

		Total *M* (*SD*) or %	Fathers *M* (*SD*) or %	Mothers *M* (*SD*) or %	Chi-Square/*t*-Statistic	*df*	*p*-Value
**Outcome**							
Parental stress		2.96 (0.46)	2.88 (0.46)	3.03 (0.46)	−4.241	701	<0.001
**Predictors**							
Social and work factors							
	Availability of assistance from family members (informal support)	4.51 (0.89)	4.48 (0.88)	4.53 (0.90)	−0.607	719	0.544
	The level of stress resulting from efforts to meet the competing demands of work and family life	2.60 (0.77)	2.62 (0.77)	2.59 (0.76)	0.383	689	0.702
	Satisfaction with work life	3.65 (0.63)	3.65 (0.62)	3.65 (0.65)	0.064	690	0.949
Endorsement of cultural values							
	Traditional attitudes toward family roles	3.47 (0.42)	3.41 (0.41)	3.53 (0.41)	−3.995	727	<0.001
	Attitudes toward traditional family values	3.13 (0.35)	3.12 (0.36)	3.15 (0.34)	−1.187	730	0.236
	Practice of filial piety (parents)	64.51 (16.07)	61.99 (16.51)	66.66 (15.40)	−3.150	462	0.002
Policy/community factors							
	Family-friendly policy (number of measures adopted)	2.18 (2.00)	2.28 (2.16)	2.08 (1.83)	1.1326	689	0.185
	Aware of the family-related activities organized by the Government or other organizations	27.1%	26.0%	28.2%	0.448	1	0.503
	Perceived effectiveness of family counseling and family education services (formal support)	2.68 (0.66)	2.71 (0.68)	2.65 (0.64)	1.159	734	0.247

**Table 3:** Associations of social, cultural, and community factors and parental stress.

		Total			Stress of Fathers			Stress of Mothers		
		*B* [95% CI]	*SE*	*p*-Value	*B* [95% CI]	*SE*	*p*-Value	*B* [95% CI]	*SE*	*p*- Value
**Model 1**										
Intercept		3.449 [3.210, 3.688]	0.122	<0.001	3.534 [3.193, 3.875]	0.173	<0.001	3.233 [2.884, 3.581]	0.177	<0.001
Demographics										
	Age	−0.009 [−0.013, −0.005]	0.002	<0.001	−0.012 [−0.017, −0.006]	0.003	<0.001	−0.005 [−0.010, 0.001]	0.003	0.105
	Monthly income	0.013 [−0.016, 0.042]	0.015	0.381	0.019 [−0.018, 0.056]	0.019	0.311	0.030 [−0.019, 0.079]	0.025	0.233
	Education level	−0.023 [−0.047, 0.001]	0.012	0.058	−0.033 [−0.064, −0.001]	0.016	0.044	−0.015 [−0.052, 0.022]	0.019	0.429
Marital status										
	Never married	0.044 [−0.226, 0.315]	0.138	0.748	0.233 [−0.174, 0.640]	0.207	0.261	−0.048 [−0.412, 0.316]	0.185	0.797
	Divorced/separated/widowed	0.219 [0.122, 0.316]	0.049	<0.001	0.311 [0.125, 0.497]	0.095	0.001	0.131 [0.012, 0.250]	0.060	0.031
	Married/cohabiting(reference)	−		−	−		−	−		−
*R* ^2^		0.056			0.091			0.021		
*F*		8.216			6.607			1.547		
*df*		5			5			5		
*n*		701			338			363		
*p*-Value		<0.001			<0.001			0.174		
**Model 2**										
Intercept		4.042 [3.693, 4.392]	0.178	<0.001	4.011 [3.501, 4.522]	0.259	<0.001	3.920 [3.439, 4.401]	0.244	<0.001
Social and work factors										
	Availability of assistance from family members (informal support)	−0.088 [−0.10, −0047]	0.021	<0.001	−0.030 [−0.093, 0.032]	0.032	0.343	−0.139 [−0.194, −0.083]	0.028	<0.001
	The level of stress resulting from efforts to meet the competing demands of work and family life	−0.044 [−0.094, 0.007]	0.026	0.090	−0.034 [−0.107, 0.040]	0.037	0.370	−0.045 [−0.115, 0.025]	0.036	0.210
	Satisfaction with work life	−0.039 [−0.101, 0.022]	0.031	0.212	−0.083 [−0.175, 0.008]	0.046	0.075	−0.008 [−0.091, 0.075]	0.042	0.850
*R* ^2^		0.097			0.104			0.108		
*F*		8.518			4.251			5.001		
*df*		8			8			8		
*n*		642			301			341		
*p*-value		<0.001			<0.001			<0.001		
**Model 3**										
Intercept		4.096 [3.284, 4.907]	0.413	<0.001	4.657 [3.467, 5.848]	0.603	<0.001	3.605 [2.495, 4.715]	0.563	<0.001
Endorsement of cultural values										
	Attitudes toward family roles	0.066 [−0.048, 0.179]	0.058	0.257	0.060 [−0.103, 0.223]	0.082	0.466	0.055 [−0.106, 0.217]	0.082	0.498
	Attitudes toward traditional family values	−0.013 [−0.146, 0.120]	0.068	0.848	−0.230 [−0.440, −0.021]	0.106	0.032	0.127 [−0.045, 0.299]	0.087	0.148
	Practice of filial piety (parents)	−0.002 [−0.005, 0.001]	0.002	0.207	−0.005 [−0.009, −0.001]	0.002	0.019	−0.001 [−0.005, 0.004]	0.002	0.812
*R* ^2^		0.108			0.156			0.159		
*F*		4.316			2.846			3.596		
*df*		11			11			11		
*n*		403			182			221		
*p*-value		<0.001			0.002			<0.001		
**Model 4**										
Intercept		3.728 [2.890, 4.566]	0.426	<0.001	4.050 [2.851, 5.249]	0.607	<0.001	3.467 [2.302, 4.633]	0.591	<0.001
Community factors										
	Family-friendly policy	0.017 [−0.007, 0.042]	0.013	0.163	0.021 [−0.013, 0.055]	0.017	0.228	0.007 [−0.029, 0.044]	0.018	0.686
	Aware of the family-related activities organized by the Government or other organizations	−0.051 [−0.160, 0.059]	0.056	0.362	−0.117 [−0.277, 0.044]	0.081	0.153	0.027 [−0.121, 0.176]	0.075	0.717
	Perceived effectiveness of family counseling and family education services (formal support)	0.132 [0.053, 0.211]	0.040	0.001	0.202 [0.092, 0.313]	0.056	<0.001	0.055 [−0.058, 0.169]	0.057	0.336
*R* ^2^		0.136			0.224			0.162		
*F*		4.311			3.432			2.817		
*df*		14			14			14		
*n*		400			181			219		
*p*-value		<0.001			<0.001			<0.001		

## Data Availability

Data are available on request from the authors.
